# Limited incremental value of balance and plantar-pressure measures beyond baseline CAIT for a 12-month composite ankle-instability outcome in competitive badminton players

**DOI:** 10.3389/fphys.2026.1918241

**Published:** 2026-08-28

**Authors:** Wangda Li

**Affiliations:** College of Physical Education, Ankang University, Ankang, Shaanxi, China

**Keywords:** ankle instability, badminton, calibration, incremental value, plantar pressure, postural control

## Abstract

**Background:**

Chronic ankle instability and recurrent symptoms are common after lateral ankle sprain, but prediction is difficult when baseline and follow-up outcomes assess overlapping instability constructs.

**Objective:**

To determine whether balance and plantar-pressure measures add predictive information beyond baseline Cumberland Ankle Instability Tool (CAIT) scores for a 12-month composite ankle-instability outcome, and to assess performance among athletes with baseline CAIT >24.

**Methods:**

In this prospective multicentre cohort, 326 badminton players were enrolled and 308 completed 12 monthly assessments. Centres 1–6 formed the development cohort (n=280), and centre 7 served as a geographic holdout (n=28). The composite outcome required repeated CAIT ≤24, giving-way or recurrent sprain, and symptoms >3 months; a new sprain was not mandatory. A six-predictor logistic model was compared with baseline CAIT, CAIT plus previous sprain, and CAIT plus the six predictors. Decision curves compared strategies. The model was refitted in athletes with baseline CAIT >24, with sensitivity analyses for index-ankle selection, training exposure, repeatability and bootstrap stability.

**Results:**

The outcome occurred in 96/280 development athletes and 11/28 holdout athletes; 68/96 and 7/11 cases, respectively, had baseline CAIT ≤24. The six-predictor model had a development AUC of 0.892 (95% CI 0.839–0.940), versus 0.951 (95% CI 0.926–0.971) for baseline CAIT, with a Brier score of 0.085. Adding the six predictors to CAIT did not improve fit (likelihood-ratio P = 0.940), and decision curves showed no consistent net-benefit gain. Among athletes with baseline CAIT >24, the refitted model had an AUC of 0.680 (95% CI 0.553–0.790) in development and 0.603 (95% CI 0.176–1.000) in the holdout. A five-predictor analysis restricted to unilateral-sprain-defined ankles yielded a development AUC of 0.921 (95% CI 0.855–0.975). Adding training volume did not materially improve fit (P = 0.085).

**Conclusion:**

Balance and plantar-pressure measures provided no clinically meaningful incremental prediction beyond baseline CAIT. High apparent performance mainly reflected persistence, recurrence or confirmation of instability already represented at baseline, rather than newly developing chronic ankle instability. The equipment-based model is not supported for pre-season screening or prevention allocation.

## 1 Introduction

Badminton involves repeated single-leg lunges, rapid deceleration, crossover steps and forefoot-dominant landings that load the lateral ankle complex (Hong et al., 2014; Lam et al., 2020). Injury-surveillance studies indicate that lower-limb injuries predominate and that the ankle is among the most frequently affected regions, with inversion mechanisms often occurring during lateral or backward steps (Liu et al., 2022; Fong et al., 2023; Pardiwala et al., 2025; Stepper et al., 2025). These movement demands make ankle function a relevant target for pre-season assessment, particularly in athletes exposed to high training volumes and repeated maximal-effort direction changes.

Chronic ankle instability (CAI) is commonly characterised by recurrent giving-way, perceived instability and repeated sprains after lateral ankle injury (Gribble et al., 2014; Doherty et al., 2016; Lin et al., 2021). It is associated with sensorimotor deficits, impaired postural stability, modified gait and landing mechanics, and reduced confidence during sport (Hertel and Corbett, 2019; Xue et al., 2024). In badminton, altered inversion control and lower-limb neuromuscular coordination during landing may contribute to recurrent symptoms and disrupted training (Zhao et al., 2025). However, CAI is a persistent clinical state rather than a simple incident event, and prospective studies must distinguish newly developing disease from persistence or recognition of instability already present at baseline.

Two measurement domains are particularly relevant. Postural-control assessments, including centre-of-pressure (COP) variables during single-leg stance and reach performance during SEBT/YBT-type tasks, quantify dynamic and static balance deficits (Hartley et al., 2018; Mohamadi et al., 2023; Phuaklikhit et al., 2024; Majlesi et al., 2025; Piri et al., 2025). Plantar-pressure mapping characterises the loading strategy beneath the foot; individuals with CAI commonly demonstrate lateralised loading, elevated lateral forefoot or rearfoot pressures and reduced medial support during walking, running or landing (Koldenhoven et al., 2016; Wan et al., 2023; Wang et al., 2025; Wen et al., 2025). Combining these domains may therefore capture both the capacity to control the body over the foot and the mechanical loading pattern applied through the index ankle.

Balance and plantar-pressure variables have seldom been integrated in a badminton-specific prediction model. Existing athlete-injury models frequently use generic outcomes, single measurement domains or machine-learning approaches that can be difficult to reproduce or interpret (Duarte Ayala et al., 2024; Zhang et al., 2024; Guo et al., 2025; Xu et al., 2026). Regression-based prediction models can provide a transparent equation, quantify calibration and discrimination, evaluate incremental value, and support cautious decision analysis when reported completely (Moons et al., 2012).

This prospective cohort study evaluated whether a limb-aligned six-predictor model combining previous lateral ankle sprain, dynamic balance, postural sway and plantar-pressure characteristics added clinically relevant predictive information beyond baseline CAIT for a 12-month composite ankle-instability outcome in competitive badminton players. The principal comparisons were baseline CAIT alone, previous-sprain history plus CAIT, the six-predictor model, and CAIT plus the six predictors. The analysis among athletes with baseline CAIT >24 was treated as a central assessment of performance in participants without CAIT-defined instability at enrolment. Additional analyses examined the index-ankle selection rule, training exposure, centre heterogeneity, nonlinear terms, age, sex, within-session repeatability and coefficient stability. The outcome was interpreted as persistent, recurrent or subsequently confirmed ankle instability rather than incident CAI.

## 2 Methods

### 2.1 Study design and participants

A prospective cohort study was conducted from September 2024 to December 2025 at seven provincial badminton training centres. The institutional review board approved the protocol. Competent adult participants provided their own written informed consent; parents or legal guardians provided consent for minors, with assent obtained where applicable. Next-of-kin consent was not used for competent adult participants. Reporting followed TRIPOD+AI recommendations, and methodological decisions were considered against PROBAST domains (Wolff et al., 2019; Collins et al., 2024). Baseline assessments were completed before the main competition period.

Eligible players were 14–30 years old, had at least three years of systematic badminton training, trained at least 10 hours per week, had no acute lower-limb injury during the preceding three months and could complete single-leg testing. Exclusion criteria were recent lower-limb surgery, neurological or vestibular disorders, uncorrected visual impairment, rehabilitation that prevented full training, refusal of consent or inability to complete follow-up. Of 412 screened athletes, 326 were enrolled. Eighteen were lost before complete 12-month data were available, leaving 308 participants with complete follow-up. Centres 1–6 contributed 280 athletes to model development; centre 7 contributed 28 athletes to a preliminary geographic holdout (**Supplementary Table 1**). Centre-specific sample sizes were 47, 47, 47, 47, 46, 46 and 28, respectively.

### 2.2 Index ankle, outcome definition and follow-up

An index ankle was defined to align participant-level predictors and outcomes. For a unilateral previous lateral ankle sprain, the previously injured ankle was selected. For bilateral previous sprain or no previous sprain, the ankle with the lower baseline CAIT score was selected; ties were resolved by selecting the non-dominant ankle. Bilateral CAIT scores, previous-sprain side, YBT side, COP side, plantar-pressure side and follow-up outcome side were retained. YBT, COP and lunge-pressure measurements corresponded to the index ankle for all 308 participants. The composite outcome was assessed on the same ankle and required all of the following: CAIT ≤24 at two consecutive monthly assessments, at least one giving-way episode or recurrent lateral ankle sprain, and subjective instability persisting for more than three months. The CAIT threshold was informed by International Ankle Consortium criteria, but baseline CAIT ≤24 alone did not define the prospective composite outcome (Gribble et al., 2014). A newly documented sprain during follow-up was not mandatory. Because lower baseline CAIT contributed to side selection in participants without a unilateral previous sprain, analyses classified the selection mechanism and included a sensitivity analysis restricted to ankles selected independently of CAIT by unilateral previous sprain.

Participants completed monthly online questionnaires for 12 months. Records included ankle-specific CAIT, giving-way frequency, new and recurrent sprains, subjective instability, symptom duration and the month when the full composite definition was first satisfied. Symptomatic cases underwent clinical confirmation by two sports-medicine physicians who were blinded to baseline biomechanical measurements. The available adjudication records contained no disagreements requiring consensus. All 308 analysed participants completed 12 monthly assessments and 365 days of follow-up; no analysed participant was censored. Consequently, the primary analysis used a fixed 12-month binary outcome rather than time-to-event modelling. Outcome-component counts and timing are reported in **Supplementary Table 2**.

### 2.3 Baseline demographic and clinical assessment

Baseline testing was completed during a single morning session at each centre to reduce fatigue and diurnal variation. Assessors recorded age, sex, height, body mass, body-mass index, dominant side, training experience, weekly training volume and side-specific previous lateral ankle sprain. Ankle status was measured bilaterally with the validated Chinese CAIT (Wang et al., 2021), and the index-ankle score was used in participant-level analyses. Weekly training volume was retained as an exposure-opportunity variable for sensitivity analysis rather than as an ankle-specific component of the primary model. The same hierarchical index-ankle rule was used for participants with bilateral or no previous sprain. Assessors received protocol training before data collection. The primary predictors contained no missing values. Documentation of assessor blinding to injury history and baseline CAIT was not available and was therefore not assumed.

### 2.4 Balance assessment

Dynamic balance was measured with the Y-Balance Test on the index ankle. Leg length was measured from the anterior superior iliac spine to the distal medial malleolus. Direction order was fixed as anterior, posteromedial and posterolateral. After three practice trials, participants completed three valid formal reaches in each direction with 20 seconds of rest between trials. The maximum valid distance in each direction was retained. Directional reach was calculated as 100 × maximum reach distance/leg length, and the composite score was calculated as 100 × (maximum anterior + maximum posteromedial + maximum posterolateral)/(3 × leg length). A trial was repeated when the stance foot moved, the reaching foot provided support or balance was not maintained.

Static balance was measured on an AMTI OR6–7 force platform at 1000 Hz. The platform was zeroed before each testing session. Participants completed one practice trial followed by three 30-second single-leg trials under eyes-open and eyes-closed conditions, with 60 seconds of rest between trials. COP signals were processed with a fourth-order zero-phase Butterworth low-pass filter at 10 Hz. Mean sway velocity, path length and 95% confidence-ellipse area were calculated for each valid trial and averaged across the three trials. The eyes-closed sway velocity represented the static-balance component of the primary model. Individual centre-specific calibration dates, stance-foot angle, arm position and visual-target distance were not retained in the protocol dataset.

### 2.5 Plantar-pressure assessment

Barefoot plantar pressure was recorded with a Novel emed-x capacitive platform at 100 Hz and 4 sensors/cm². The system was zeroed before each session; individual centre-specific calibration dates were not retained. Quiet standing comprised three 30-second trials with 60 seconds of rest. For the sport-specific forehand lunge, the participant used a racket, contacted the platform with the index ankle as the leading foot, aimed for a target distance approximating body height, and maintained the foot angle near 0°. Three practice attempts preceded five formal lunge trials, with 60 seconds of rest. No instrumented approach-velocity constraint was available; this procedural limitation was considered when interpreting ecological validity. A common regional-mask definition was applied in the supplied analysis dataset.

The lateral forefoot peak-pressure index was the mean across five valid lunge trials of the maximum instantaneous pressure in metatarsal regions M4-M5. The medial-to-lateral plantar load ratio was the mean medial force-time integral divided by the mean lateral force-time integral; medial regions comprised the hallux, M1-M2, medial midfoot and medial heel, whereas lateral regions comprised M3-M5, lateral midfoot and lateral heel. Heel contact-area asymmetry was calculated from the mean left and right heel areas across three quiet-standing trials as 100 × |Aindex - Acontralateral|/[(Aindex + Acontralateral)/2]. Trial-level data, masks and operational definitions are detailed in **Supplementary Methods** and **Supplementary Table 3**. Within-session repeatability was quantified from the formal trials, but it was not interpreted as evidence of between-day, inter-rater or cross-centre measurement equivalence.

### 2.6 Sample-size considerations

Sample-size adequacy was assessed using contemporary criteria for binary-outcome prediction models (Riley et al., 2019). With six predictor parameters, an outcome proportion of 0.343, an anticipated Cox–Snell R² of 0.20 and target global shrinkage of 0.90, approximately 239 participants were required to limit overfitting; approximately 347 were required to estimate the overall outcome proportion with a 95% confidence interval half-width of 0.05. The development cohort of 280 participants and 96 events met the shrinkage-based criterion but not the more conservative overall-risk precision criterion. The model was therefore treated as exploratory, and the 28-participant holdout was interpreted only as a preliminary geographic evaluation rather than definitive external validation.

### 2.7 Statistical analysis and model construction

Analyses were reproduced in Python 3.13 using NumPy, SciPy, pandas, statsmodels and scikit-learn. Continuous variables are summarised as mean ± standard deviation and categorical variables as counts and percentages. Development-cohort comparisons used Welch t tests for continuous variables and chi-square or Fisher exact tests for categorical variables. The primary predictors, baseline CAIT and training-volume variable had no missing observations; analyses were therefore complete-case analyses without imputation. Two-sided P values were interpreted descriptively because the study evaluated prediction rather than causal effects.

The primary logistic model contained six clinically specified variables representing the study hypothesis: previous lateral ankle sprain, YBT composite score, eyes-closed COP sway velocity, lateral forefoot peak pressure, medial-to-lateral load ratio and heel contact-area asymmetry. The binary sprain variable was coded 0/1, and continuous variables were standardised using development-cohort means and standard deviations. No univariable screening, LASSO selection or backward elimination was applied. Weekly training volume was not included in the primary equation because it represents exposure opportunity rather than an ankle-specific functional phenotype and would increase model dimensionality; it was added in a dedicated sensitivity model. Separate sensitivity models added body mass or BMI. Variance inflation factors were calculated only to describe linear collinearity. The complete equation, intercept, coding and standardisation constants are provided in **Supplementary Table 4**.

Discrimination was quantified by AUC with stratified-bootstrap confidence intervals. Overall accuracy was quantified by Brier score and compared with the null-model Brier score. Calibration was assessed graphically and by calibration-in-the-large, calibration slope and observed-to-expected ratio with 95% confidence intervals; the Hosmer-Lemeshow test was not used as evidence of calibration (Van Calster et al., 2019). Internal validation repeated model fitting and standardisation in 2000 bootstrap samples to estimate optimism. Locked coefficients were applied to centre 7, and leave-one-centre-out internal-external cross-validation evaluated heterogeneity across all seven centres; the small holdout was interpreted in light of sample-size recommendations for model evaluation (Riley et al., 2021). Baseline CAIT alone, CAIT plus previous sprain, the six-predictor model and CAIT plus the six predictors were evaluated with the same discrimination, Brier and calibration measures. Sensitivity and specificity were calculated at the clinically established CAIT ≤24 threshold. Decision-curve analysis compared these four strategies directly over threshold probabilities of 0.10-0.50 (Vickers and Elkin, 2006). In the central baseline-CAIT>24 analysis, standardisation and model fitting were repeated within the restricted development cohort and the resulting locked equation was applied to the restricted holdout. Index-ankle assignment was classified as unilateral previous sprain, lower baseline CAIT or non-dominant-side tie-break; a five-predictor model excluding the constant sprain term was fitted in the unilateral-sprain subgroup. Additional analyses added training volume, body mass or BMI, examined nonlinear terms and selected interactions, added centre effects, and explored sex and age strata. Within-session repeatability was described by two-way mixed-effects absolute-agreement ICC(A,1) and within-subject coefficient of variation. Coefficient stability was summarised from 2000 complete bootstrap refits by percentile odds-ratio intervals and the proportion of refits retaining the adverse-direction sign.

## 3 Results

### 3.1 Participant flow and baseline characteristics

**Figure 1:**
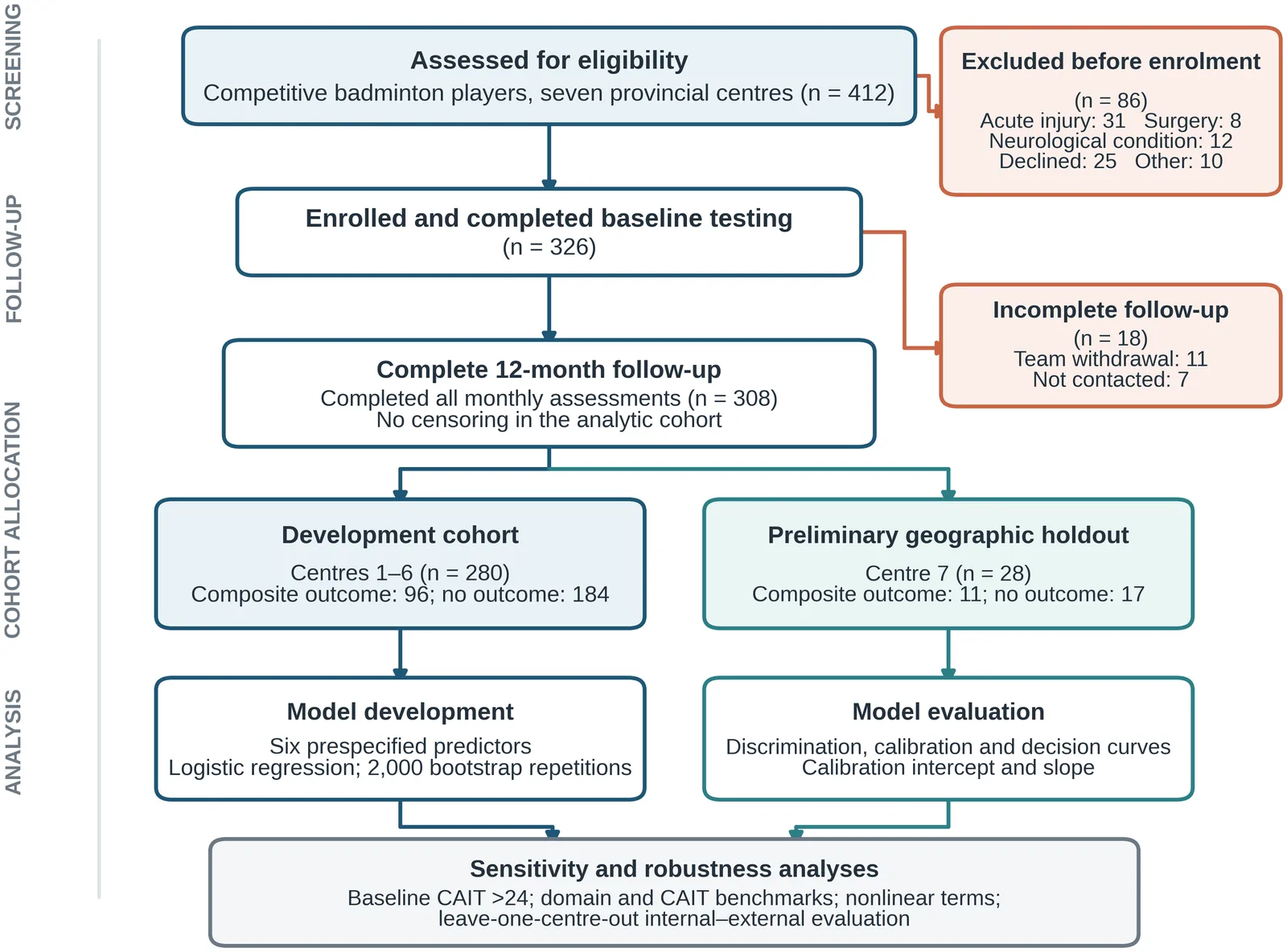
Participant enrolment, complete follow-up, cohort allocation, model development, preliminary geographic evaluation and sensitivity analyses.

Of 412 screened players, 326 underwent baseline testing and 308 completed all monthly assessments (**Figure 1**). The development cohort included 280 athletes, of whom 96 (34.3%) met the composite outcome; the geographic holdout included 28 athletes, of whom 11 (39.3%) met the outcome. Across the full cohort, 107 participants met the complete composite definition, 77 after a newly documented sprain and 30 without a new sprain. Fourteen participants met only the repeated-CAIT component, 20 met only the giving-way/recurrent-sprain component and five met CAIT plus an event but had symptoms for no more than three months. Eight outcome-positive participants had bilateral findings; every outcome-positive participant had involvement of the index ankle. Index-ankle assignment was based on unilateral previous sprain in 109 participants, lower baseline CAIT in 198 and the non-dominant-side tie-break in one; corresponding development-cohort counts were 101, 179 and zero.

Development-cohort characteristics are shown in **Table 1**. Baseline CAIT was ≤24 in 68/96 (70.8%) outcome-positive athletes and 8/184 (4.3%) outcome-negative athletes. Across all 308 athletes, the composite outcome occurred in 69/129 (53.5%) with previous lateral ankle sprain and 38/179 (21.2%) without previous sprain; it occurred in 75/83 (90.4%) with baseline CAIT ≤24 and 32/225 (14.2%) with baseline CAIT >24. These patterns show that the high overall outcome frequency reflected a broad composite definition and a substantial burden of instability already represented at baseline. The pooled cohort therefore combined distinct tasks, including persistence of existing symptoms, recurrence after previous sprain and newly developing or newly recognised instability.

**Table 1:** Baseline demographic, training and clinical characteristics of the development cohort.

Characteristic	Composite-outcome group (n = 96)	No-outcome group (n = 184)	P value
Age (years), mean ± SD	19.4 ± 2.6	19.7 ± 2.4	0.348
Sex, male/female, n	54/42	98/86	0.726
Height (cm)	172.4 ± 8.1	171.9 ± 7.6	0.617
Body mass (kg)	68.7 ± 9.4	66.2 ± 8.6	0.031
Body-mass index (kg/m²)	23.1 ± 2.4	22.3 ± 2.1	0.012
Training experience (years)	7.8 ± 3.1	7.4 ± 2.8	0.291
Training volume (h/week)	18.6 ± 4.7	16.2 ± 4.1	< 0.001
Previous lateral ankle sprain, n (%)	64 (66.7)	57 (31.0)	< 0.001
Bilateral previous sprain, n (%)	10 (10.4)	10 (5.4)	0.196
Index ankle, right, n (%)	32 (33.3)	61 (33.2)	1.000
Baseline CAIT score	21.4 ± 3.2	27.6 ± 1.9	< 0.001
Baseline CAIT ≤24, n (%)	68 (70.8)	8 (4.3)	< 0.001

### 3.2 Balance and plantar-pressure characteristics by outcome

**Figure 2:**
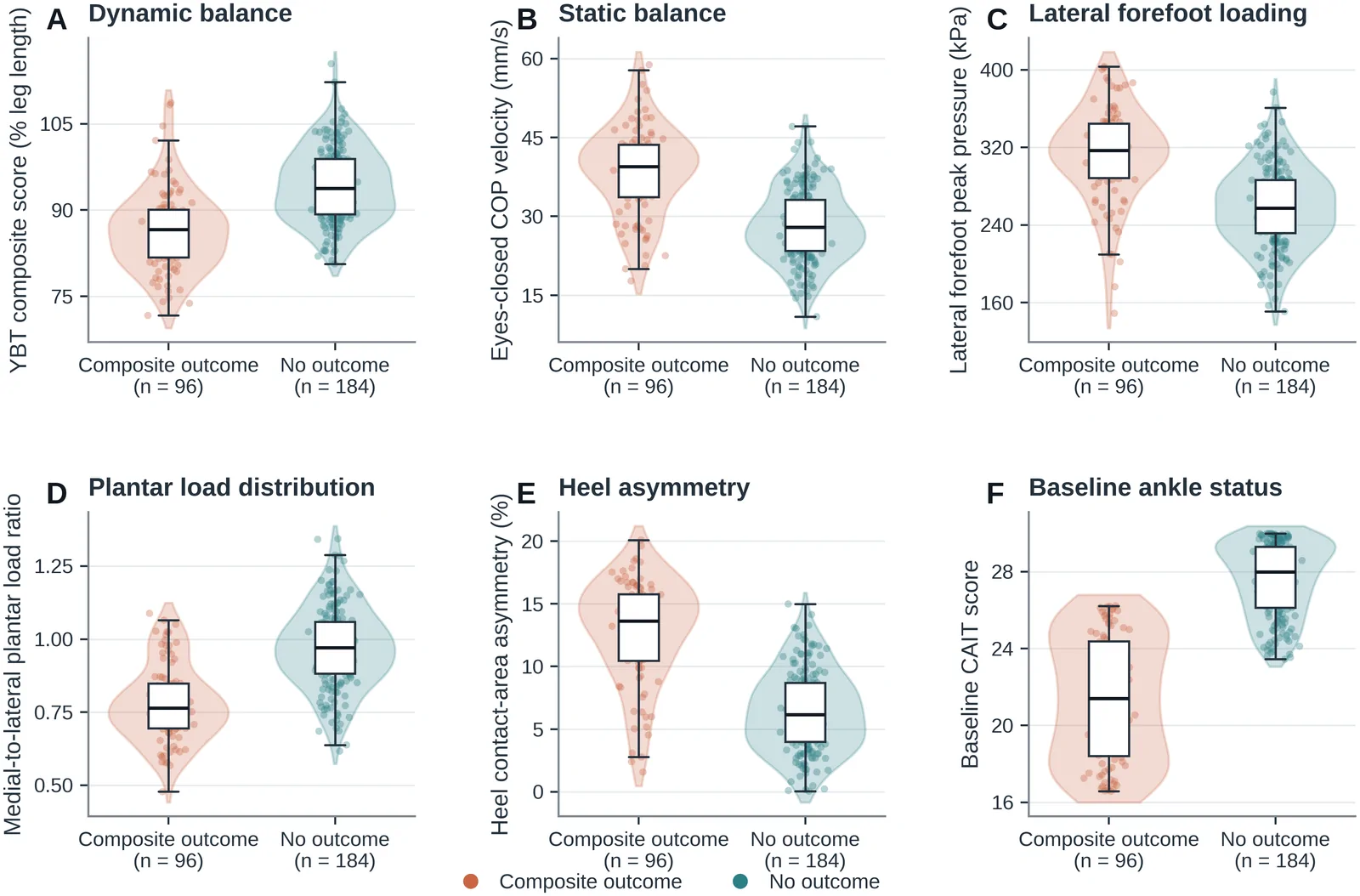
Violin plots with embedded box plots and participant-level observations showing baseline distributions in the development cohort. All descriptive between-group P values were <0.001. **(A)** Dynamic balance, **(B)** Static balance, **(C)** Lateral forefoot loading, **(D)** Plantar load distribution, **(E)** Heel asymmetry, and **(F)** Baseline ankle status.

Baseline balance and plantar-pressure variables differed between outcome groups (**Table 2**). Outcome-positive athletes had lower YBT composite and directional reach scores, higher eyes-open and eyes-closed COP sway velocity, larger COP ellipse area and path length, higher lateral forefoot pressure, lower medial forefoot pressure, a lower medial-to-lateral load ratio and greater heel contact-area asymmetry. **Figure 2** displays the distributions of the five biomechanical predictors and baseline CAIT. These baseline differences were treated as predictive associations with a composite outcome that substantially overlapped baseline instability status; they were not interpreted as evidence of incident disease or independent biomechanical mechanisms.

**Table 2:** Baseline balance-function and plantar-pressure parameters in the development cohort.

Variable	Composite-outcome group (n = 96)	No-outcome group (n = 184)	t	P value
YBT composite score (% leg length)	86.4 ± 7.1	94.2 ± 6.4	−9.02	< 0.001
YBT anterior reach (% LL)	63.2 ± 6.8	70.4 ± 5.9	−8.74	< 0.001
YBT posteromedial reach (% LL)	97.8 ± 8.1	104.6 ± 7.4	−6.82	< 0.001
YBT posterolateral reach (% LL)	98.1 ± 7.7	107.6 ± 7.2	−10.00	< 0.001
COP sway velocity, eyes open (mm/s)	22.4 ± 5.6	17.8 ± 4.8	6.84	< 0.001
COP sway velocity, eyes closed (mm/s)	38.6 ± 8.4	28.3 ± 6.9	10.33	< 0.001
95% COP sway-ellipse area (mm²)	318.4 ± 76.5	241.7 ± 58.4	8.60	< 0.001
Lateral forefoot peak pressure (kPa)	312.8 ± 48.7	258.3 ± 41.2	9.36	< 0.001
Medial forefoot peak pressure (kPa)	241.2 ± 43.5	266.4 ± 39.6	−4.74	< 0.001
Medial-to-lateral plantar load ratio	0.78 ± 0.13	0.97 ± 0.14	−11.30	< 0.001
Heel contact-area asymmetry (%)	12.8 ± 4.1	6.4 ± 3.2	13.32	< 0.001
Centre-of-pressure path length (mm)	482.1 ± 87.4	392.6 ± 73.5	8.58	< 0.001

Trial-level aggregation reproduced the participant-level values used in analysis. Within-session ICC(A,1) values were 0.994 for the YBT trial-composite, 0.991 for eyes-closed COP velocity, 0.964 for lateral forefoot pressure, 0.982 for the medial-to-lateral ratio and 0.957 for heel asymmetry; corresponding within-subject coefficients of variation were 0.7%, 2.6%, 3.5%, 2.5% and 11.4%. These estimates describe repeatability during the same session only and do not establish between-day, inter-rater or cross-centre reliability. The index ankle was used for all YBT, COP and lunge-pressure variables, and the outcome involved the index ankle in all 107 cases.

### 3.3 Primary six-predictor model

The six clinically specified predictors were retained in the primary model without data-driven elimination (**Table 3**). The model odds ratios for adverse one-standard-deviation changes were 3.42 (95% CI 1.51-7.73) for previous lateral ankle sprain, 2.17 (1.31-3.59) for lower YBT composite score, 1.95 (1.19-3.22) for higher eyes-closed COP sway velocity, 1.73 (1.05-2.85) for higher lateral forefoot peak pressure, 2.04 (1.24-3.37) for lower medial-to-lateral load ratio and 1.61 (0.85-3.04) for higher heel asymmetry. Across 2000 bootstrap refits, adverse-direction signs were retained in 99.8%, 99.7%, 99.9%, 97.6%, 100.0% and 93.7% of samples, respectively; the bootstrap interval for heel asymmetry remained imprecise. These coefficients quantify multivariable prediction contributions and were not interpreted as causal, independently modifiable mechanisms. Variance inflation factors ranged from 1.07 to 2.68.

**Table 3:** Logistic regression coefficients for the primary 12-month composite ankle-instability model.

Predictor	β (SE)	Wald χ²	Model OR (95% CI)	P value
Previous lateral ankle sprain (yes vs no)	1.230 (0.416)	8.75	3.42 (1.51–7.73)	0.003
YBT composite score (per 1-SD decrease)	0.776 (0.257)	9.14	2.17 (1.31–3.59)	0.003
COP velocity, eyes closed (per 1-SD increase)	0.670 (0.255)	6.91	1.95 (1.19–3.22)	0.009
Lateral forefoot peak pressure (per 1-SD increase)	0.550 (0.254)	4.69	1.73 (1.05–2.85)	0.030
Medial-to-lateral load ratio (per 1-SD decrease)	0.713 (0.255)	7.81	2.04 (1.24–3.37)	0.005
Heel contact-area asymmetry (per 1-SD increase)	0.478 (0.324)	2.18	1.61 (0.85–3.04)	0.140

The fitted equation used an intercept of −1.604, a coefficient of 1.230 for previous sprain coded 1 versus 0, and standardised continuous-variable coefficients of −0.776 for YBT, 0.670 for eyes-closed COP velocity, 0.550 for lateral forefoot pressure, −0.713 for the medial-to-lateral ratio and 0.478 for heel asymmetry. The full equation and standardisation constants are supplied in **Supplementary Table 4** to permit independent calculation.

### 3.4 Incremental value and benchmark analyses

**Figure 3:**
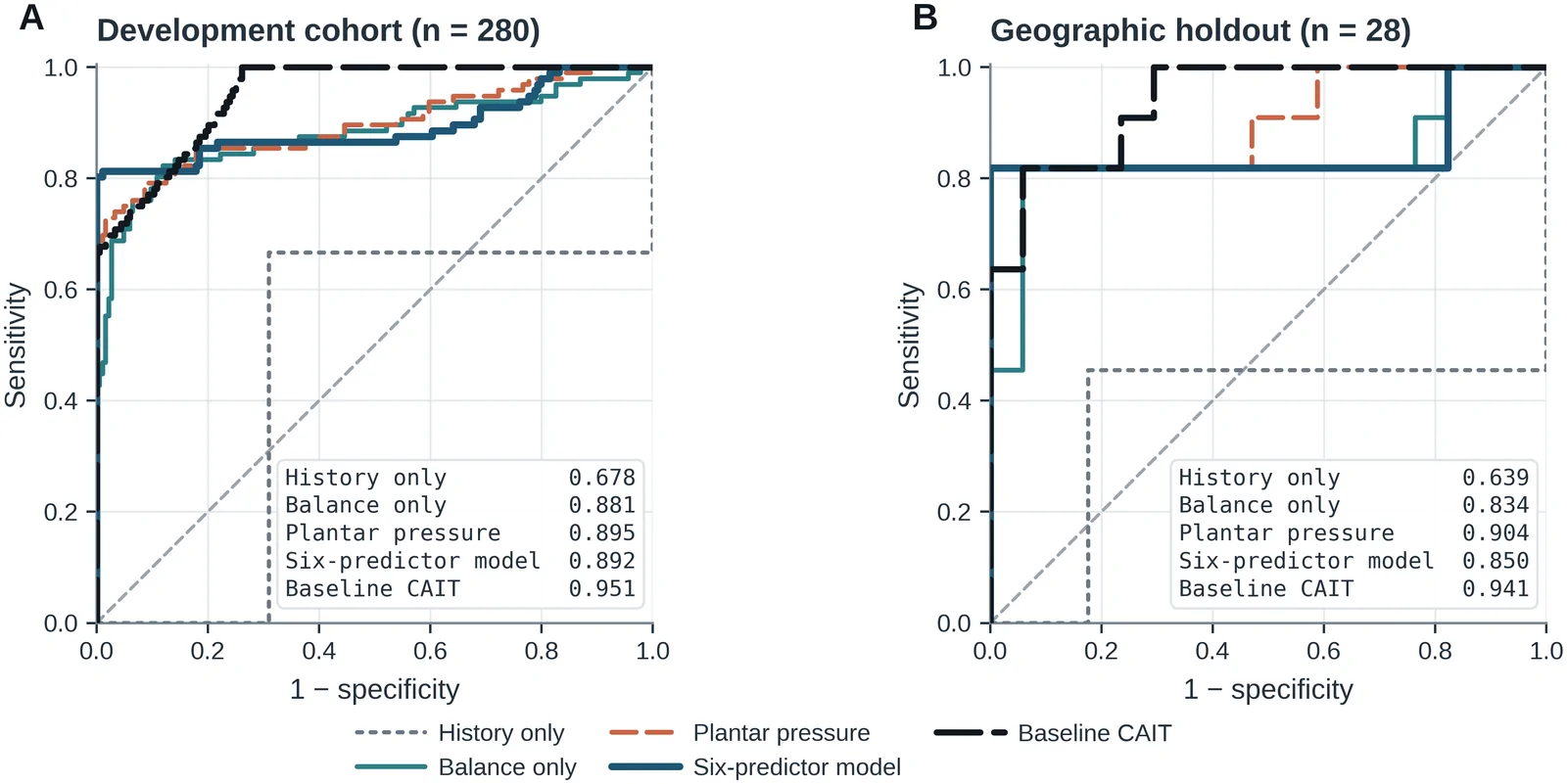
Receiver-operating-characteristic curves comparing history-only, balance-only, plantar-pressure, six-predictor and baseline-CAIT models in the development cohort and preliminary geographic holdout. **(A)** Development cohort, **(B)** Geographic holdout.

Domain-model comparisons are summarised in **Table 4** and **Figure 3**. In development data, the history-only, balance-only, plantar-pressure and combined six-predictor models had AUCs of 0.678, 0.881, 0.895 and 0.892, respectively, with Brier scores of 0.199, 0.113, 0.100 and 0.080. Baseline CAIT alone had an AUC of 0.951 (95% CI 0.926-0.971), Brier score of 0.085 (95% CI 0.065-0.105), calibration-in-the-large of 0.000 (95% CI -0.419 to 0.419), calibration slope of 1.000 (95% CI 0.729-1.271) and observed-to-expected ratio of 1.00 (95% CI 0.81-1.22). In the holdout, CAIT had an AUC of 0.941 (95% CI 0.845-1.000), Brier score of 0.092, calibration-in-the-large of 0.319, slope of 1.280 and observed-to-expected ratio of 1.08, all with wide intervals. At CAIT ≤24, development sensitivity was 0.708 (95% CI 0.611-0.790) and specificity was 0.957 (0.917-0.978); holdout sensitivity was 0.636 (0.354-0.848) and specificity was 1.000 (0.816-1.000). Adding previous sprain to CAIT did not improve fit (P = 0.662), and adding all six predictors produced an AUC of 0.952 but no likelihood-ratio improvement over CAIT alone (P = 0.940). Decision curves showed no consistent net-benefit advantage for the equipment-based model or the augmented CAIT models over baseline CAIT.

**Table 4:** Discrimination, overall accuracy, calibration and central comparative analyses.

Analysis	n/events	AUC (95% CI)	Brier score	Calibration/comparison
Six-predictor model: development	280/96	0.892 (0.839-0.940)	0.080	CITL 0.000; slope 1.000
Six-predictor model: bootstrap corrected	280/96	0.882	0.086	2,000 complete refits
Six-predictor model: geographic holdout	28/11	0.850 (0.636-1.000)	0.094	CITL 0.187; slope 0.789
Leave-one-centre-out pooled evaluation	308/107	0.879	0.088	CITL -0.069; slope 0.915
History-only model: development	280/96	0.678 (0.623-0.735)	0.199	Reference domain
Balance-only model: development	280/96	0.881 (0.828-0.930)	0.113	LR vs combined P < 0.001
Pressure-only model: development	280/96	0.895 (0.846-0.938)	0.100	LR vs combined P < 0.001
Baseline CAIT alone: development	280/96	0.951 (0.926-0.971)	0.085	CITL 0.000; slope 1.000; O:E 1.00
Baseline CAIT alone: holdout	28/11	0.941 (0.845-1.000)	0.092	CITL 0.319; slope 1.280; O:E 1.08
CAIT plus previous sprain: development	280/96	0.950 (0.926-0.971)	0.085	LR vs CAIT P = 0.662
CAIT plus six predictors: development	280/96	0.952 (0.930-0.971)	0.086	LR vs CAIT P = 0.940
Baseline CAIT >24: refitted development	204/28	0.680 (0.553-0.790)	0.099	CITL 0.000; slope 1.000; O:E 1.00
Baseline CAIT >24: restricted holdout	21/4	0.603 (0.176-1.000)	0.130	CITL 0.249; slope 0.714; O:E 1.19

### 3.5 Central subgroup, robustness and sensitivity analyses

**Figure 4:**
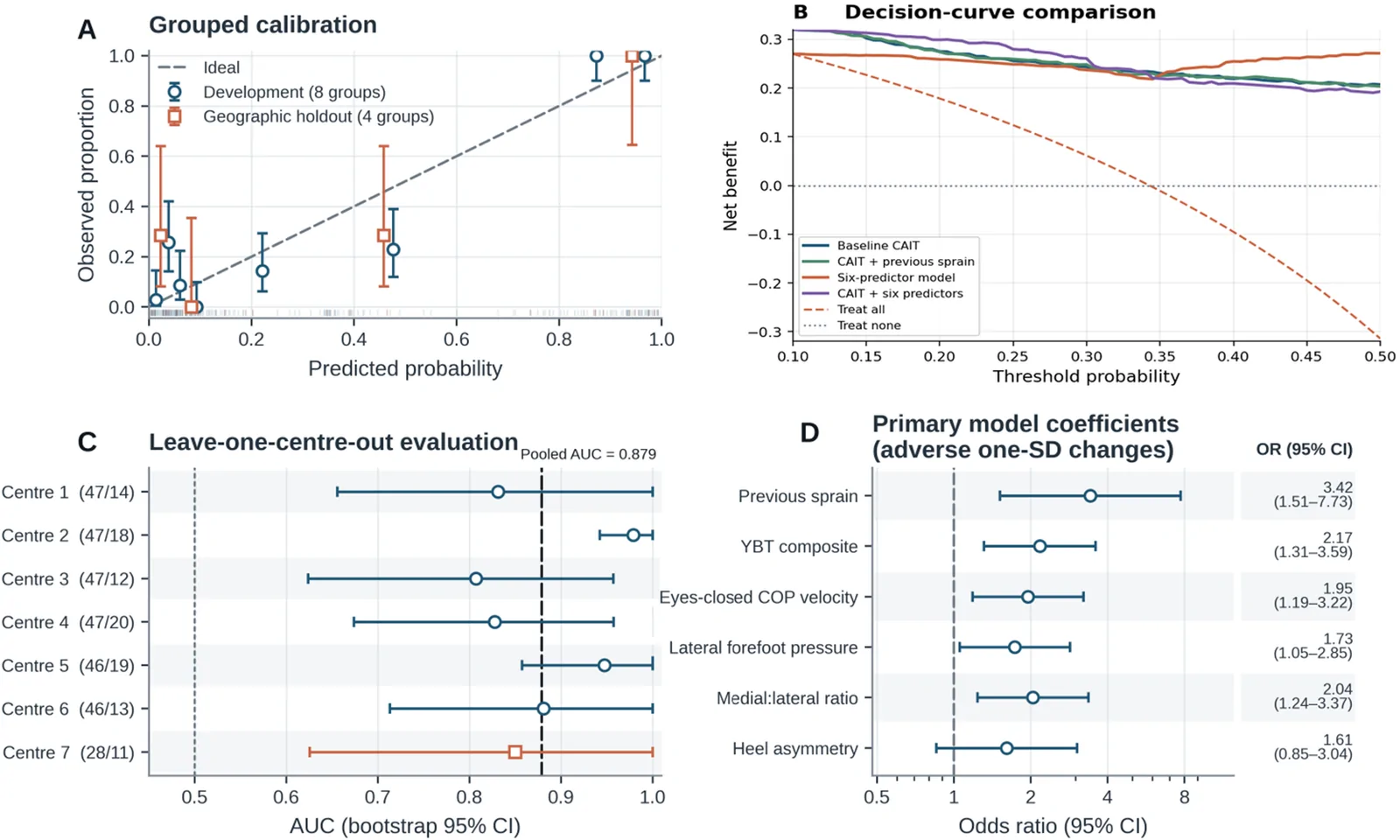
Primary and comparative model performance. **(A)** Grouped calibration plots for the development cohort and geographic holdout. **(B)** Development-cohort decision curves directly comparing baseline CAIT, CAIT plus previous sprain, the six-predictor model, CAIT plus the six predictors, treat-all and treat-none strategies. **(C)** Leave-one-centre-out AUCs with stratified-bootstrap 95% confidence intervals; labels report n/events. **(D)** Primary-model coefficients expressed as odds ratios for adverse one-standard-deviation changes.

Primary model calibration, decision-curve, leave-one-centre-out and coefficient results are summarised in **Figure 4**. The primary six-predictor model had an apparent development AUC of 0.892 (95% CI 0.839-0.940) and Brier score of 0.080, compared with a null-model Brier score of 0.225. After 2000 bootstrap repetitions, the optimism-corrected AUC was 0.882 and the corrected Brier score was 0.086. Development calibration-in-the-large was 0.000 (95% CI -0.389 to 0.389), the calibration slope was 1.000 (95% CI 0.781-1.219) and the observed-to-expected ratio was 1.00 (95% CI 0.81-1.22). Applying the locked equation to centre 7 yielded an AUC of 0.850 (95% CI 0.636-1.000), Brier score of 0.094, calibration-in-the-large of 0.187 (95% CI -1.060 to 1.435), calibration slope of 0.789 (95% CI 0.257-1.322) and observed-to-expected ratio of 1.04 (95% CI 0.52-1.87). These estimates demonstrate technical application of the equation but are too imprecise to establish geographic reproducibility.

In the central analysis restricted to baseline CAIT >24, refitting the six-predictor model in 204 development athletes with 28 outcomes produced an AUC of 0.680 (95% CI 0.553-0.790), Brier score of 0.099, calibration-in-the-large of 0.000 (95% CI -0.426 to 0.426), calibration slope of 1.000 (0.545-1.455) and observed-to-expected ratio of 1.00 (0.66-1.45). Application to 21 holdout athletes with four outcomes produced an AUC of 0.603 (95% CI 0.176-1.000), Brier score of 0.130, calibration-in-the-large of 0.249 (95% CI -0.943 to 1.442), calibration slope of 0.714 (-0.381 to 1.809) and observed-to-expected ratio of 1.19 (0.32-3.04). Development AUCs were similarly limited among participants with and without previous sprain (0.666 and 0.670). In the 101 development participants whose index ankle was determined independently of CAIT by unilateral previous sprain, a five-predictor model had an apparent AUC of 0.921 (95% CI 0.855-0.975) and Brier score of 0.091. This was the closest available CAIT-independent sensitivity analysis, but alternative-side biomechanical measurements were not collected; consequently, it neither eliminated nor quantified the bias introduced by the index-ankle selection rule. Adding weekly training volume yielded an odds ratio of 1.43 per standard deviation (95% CI 0.95-2.15), development AUC of 0.904, Brier score of 0.079 and likelihood-ratio P = 0.085. Development calibration-in-the-large was 0.000 (95% CI -0.392 to 0.392), the calibration slope was 1.000 (0.778-1.222) and the observed-to-expected ratio was 1.00 (0.81-1.22). In the holdout, calibration-in-the-large was 0.096 (-1.195 to 1.387), the slope was 0.729 (0.235-1.223) and the observed-to-expected ratio was 1.02 (0.51-1.83), compared with 0.187, 0.789 and 1.04 for the primary model. The six primary odds ratios changed by -6.1% to +3.3% (mean absolute change 2.6%). Separate additions of body mass or BMI also produced no material improvement. Leave-one-centre-out AUCs ranged from 0.807 to 0.979, but this analysis assessed predictive heterogeneity rather than measurement equivalence. The probability cutoff of 0.34 was data-derived and was not interpreted as an actionable clinical threshold.

## 4 Discussion

The central finding of this study is that objective balance and plantar-pressure measurements did not add clinically meaningful prediction beyond baseline CAIT for the stated 12-month composite ankle-instability outcome. Although the six-predictor model had an apparent AUC of 0.892 and an optimism-corrected AUC of 0.882, the outcome should not be interpreted as incident CAI: a new sprain was not required, 70.1% of all outcome-positive athletes had baseline CAIT ≤24, and 90.4% of participants with baseline CAIT ≤24 later met the composite definition. The model therefore primarily characterises persistence, recurrence or subsequent confirmation of instability already represented at baseline.

Baseline CAIT was the strongest and least burdensome benchmark. CAIT alone achieved a development AUC of 0.951 with good apparent calibration, while adding previous injury, balance and plantar-pressure measurements did not improve model fit. The direct decision-curve comparison likewise showed no consistent gain over CAIT. Most importantly, when the analysis was restricted to athletes with baseline CAIT >24 and the model was refitted, discrimination decreased to 0.680 in development and 0.603 in the very small holdout. Similar AUCs in the prior-sprain and no-prior-sprain strata indicate limited ability to distinguish newly emerging instability in either subgroup. A clinically meaningful 12-month prediction task is therefore not established for athletes without baseline CAIT-defined instability.

The directions of the balance and plantar-pressure coefficients were biomechanically coherent with reports of reduced reach performance, faster postural sway and lateralised loading in CAI populations (Koldenhoven et al., 2016; Hartley et al., 2018; Mohamadi et al., 2023; Wan et al., 2023; Phuaklikhit et al., 2024; Majlesi et al., 2025; Piri et al., 2025; Wang et al., 2025; Wen et al., 2025). Bootstrap refits showed generally stable coefficient directions, whereas heel asymmetry remained less precise. This stability concerns the fitted prediction equation, not causality or independent biological mechanisms. The baseline measurements may be concurrent manifestations of existing instability, and the outcome-related limb-selection procedure further limits mechanistic interpretation.

The negative incremental-value result determines the practical interpretation. The six-predictor model requires a force platform, a plantar-pressure system, trained assessors and standardised processing, yet it did not improve on a brief questionnaire. Decision-curve differences between CAIT and the more complex strategies were small and inconsistent, and the study did not establish an actionable threshold or evaluate model-guided intervention. Accordingly, the model should not be presented as a sideline screen, an individualised clinical calculator or a basis for preventive-resource allocation.

The index-ankle rule selected 109 ankles by unilateral previous sprain, 198 by lower baseline CAIT and one by the non-dominant tie-break. The high AUC in the unilateral-sprain subgroup indicates that predictive separation was not confined to CAIT-selected ankles, but alternative-side YBT, COP and lunge-pressure measurements were not collected. The subgroup analysis therefore cannot eliminate or quantify selection bias or determine performance on a prespecified side independent of CAIT. Weekly training volume was associated with greater exposure opportunity, but its addition did not materially alter model fit, calibration or the biomechanical coefficients: the primary odds ratios changed by no more than 6.1%, and holdout calibration remained highly imprecise (calibration-in-the-large 0.096, slope 0.729 and observed-to-expected ratio 1.02). Within-session repeatability was high for most measures, although heel-asymmetry variability was greater; these same-session estimates do not compensate for the absence of independent between-day, inter-rater or cross-centre reliability studies.

Centre 7 should be regarded only as a technical geographic holdout. Its 28 participants and 11 outcomes, and particularly the four outcomes in the baseline-CAIT>24 subgroup, are far below requirements for precise model evaluation (Riley et al., 2021). The wide discrimination and calibration intervals cannot support a claim that performance was geographically reproduced. The cohort also combines adolescents and adults, athletes with and without previous sprain, and participants with and without baseline CAIT-defined instability. These groups represent different clinical tasks and should be evaluated in separate, adequately powered cohorts, particularly athletes without baseline CAI and athletes recovering from a documented acute lateral ankle sprain.

Balance, strength, landing-retraining and neuromuscular programmes remain plausible interventions for athletes with ankle instability, but this cohort did not compare model-guided prevention with universal training or CAIT-based screening. Evidence that mobility interventions can alter ankle dorsiflexion while considering performance (Patti et al., 2025), and that functional rehabilitation can improve dynamic balance in CAI (Ahern et al., 2021), supports prospective intervention research. It does not establish that a probability cutoff of 0.34 is clinically justified or that changing the model score will prevent the composite outcome.

Several limitations remain. The composite outcome incorporated repeated low CAIT, giving-way or recurrent sprain and symptom persistence, so it substantially overlapped baseline ankle status. Baseline giving-way history was not available as a structured enrolment variable, and mechanical instability was not evaluated by imaging or stress testing. The outcome-related index-side rule could not be fully evaluated because alternative-side biomechanical measurements were unavailable. The holdout was extremely small, the cohort came from one country, and subgroup estimates were imprecise. Separate between-day and inter-rater reliability data were unavailable; centre-specific calibration dates and static-stance details for arm position, visual-target distance and foot angle were not retained. Barefoot pressure testing may not generalise to badminton footwear, approach velocity was not instrumented, and assessor blinding to clinical history was undocumented. Baseline weekly training volume does not capture time-varying exposure, recovery, footwear or preventive exercise. Larger prospective studies should use clinically homogeneous target populations, define the analysed ankle independently of the outcome measure, compare simple clinical benchmarks, and test calibration and clinical impact in independent settings.

## 5 Conclusion

In this prospective cohort, balance and plantar-pressure measures did not provide incremental predictive value beyond baseline CAIT for a 12-month composite ankle-instability outcome. The apparent model performance was largely associated with persistence, recurrence or subsequent confirmation of instability already present at baseline, and discrimination was limited among athletes with baseline CAIT >24. The very small geographic holdout supports only technical application of the equation. The equipment-based model should not be used for pre-season screening, individual risk calculation or prevention allocation without new, limb-specific independent studies in clearly defined target populations.

## Data Availability

The individual-level dataset is not publicly available because of institutional privacy restrictions. A data dictionary, operational predictor definitions, complete model coefficients and reproducible analysis code accompany the submission. Deidentified data sufficient for verification may be made available by the corresponding author on reasonable request and subject to institutional and ethics approval. Requests to access the datasets should be directed to Wangda Li, 15961708232@163.com.
