# Supplementary material for: Limited incremental value of balance and plantar-pressure measures beyond baseline CAIT for a 12-month composite ankle-instability outcome in competitive badminton players
Source: Front Physiol. 2026 Aug 28;17:1918241. doi: 10.3389/fphys.2026.1918241 (PMC13561929; doi:10.3389/fphys.2026.1918241)
Supplement: Supplementary file 1 [file SupplementaryFile1.docx]

**Supplementary Material**

# Supplementary Methods

## Index-ankle construction and side consistency

The analysis used one index ankle per participant. A unilateral previously sprained ankle was selected first; otherwise the ankle with the lower baseline CAIT was selected, with the non-dominant ankle used for ties. Previous-sprain side, bilateral baseline CAIT, YBT side, COP side, lunge-pressure side and outcome side were retained. Across 308 participants, index-ankle assignment was based on unilateral previous sprain in 109, lower baseline CAIT in 198 and the non-dominant-side tie-break in one. All participants had YBT, COP and lunge-pressure measurements aligned to the index ankle, and the index ankle was involved in all 107 composite outcomes. The CAIT-independent sensitivity analysis included 101 development participants whose index ankle was selected by unilateral previous sprain; because previous sprain was constant in this subgroup, the fitted model contained the five continuous biomechanical predictors. Alternative-side YBT, COP and lunge-pressure data were not collected, so this subgroup analysis was the closest available CAIT-independent assessment but could not eliminate or quantify the selection bias or support a complete ankle-level analysis.

## Outcome algorithm

The full composite outcome required, on the same ankle, CAIT ≤24 at two consecutive monthly assessments, at least one giving-way episode or recurrent sprain, and subjective instability for at least four consecutive monthly assessments. A newly documented sprain was not required. The first month when all three components had been accumulated was recorded as the confirmation month. Monthly records were available for 12 months for both ankles of every analysed participant (7392 ankle-month records).

## Measurement protocols and operational formulas

YBT leg length was measured from the anterior superior iliac spine to the distal medial malleolus. The fixed direction order was anterior, posteromedial and posterolateral. Testing was completed after three practice trials, with three formal trials per direction and 20 seconds of rest. COP testing used an AMTI OR6-7 force platform at 1000 Hz, zeroing before each session, one practice trial and three 30-second formal trials for each visual condition, with 60 seconds of rest and a fourth-order zero-phase Butterworth low-pass filter at 10 Hz. Plantar-pressure testing used a Novel emed-x platform at 100 Hz and 4 sensors/cm², zeroing before each session, three quiet-standing trials and five formal lunge trials after three practice attempts, with 60 seconds of rest. The lunge was barefoot, racket-assisted and directed to a target approximating body height with the foot angle near 0°. Trial-level within-session repeatability was quantified by ICC(A,1) and within-subject coefficient of variation. Separate between-day, inter-rater and cross-centre reliability testing was not performed.

Lateral forefoot peak pressure = mean across five valid lunge trials of the maximum instantaneous pressure in M4–M5. Medial-to-lateral load ratio = mean force-time integral in the hallux, M1–M2, medial midfoot and medial heel divided by the mean force-time integral in M3–M5, lateral midfoot and lateral heel. Heel contact-area asymmetry (%) = 100 × |Aindex − Acontralateral|/[(Aindex + Acontralateral)/2], where areas are averages across three quiet-standing trials.

# Table S1. Development and geographic-holdout characteristics

| **Characteristic** | **Development (n = 280)** | **Holdout (n = 28)** | **P value** |
| --- | --- | --- | --- |
| Age (years) | 19.60 ± 2.47 | 19.50 ± 2.53 | 0.853 |
| Height (cm) | 172.07 ± 7.76 | 171.85 ± 6.63 | 0.868 |
| Body mass (kg) | 67.06 ± 8.94 | 68.29 ± 9.61 | 0.521 |
| BMI (kg/m²) | 22.60 ± 2.23 | 23.06 ± 2.31 | 0.319 |
| Training experience (years) | 7.54 ± 2.91 | 8.05 ± 2.91 | 0.382 |
| Training volume (h/week) | 17.02 ± 4.46 | 18.49 ± 4.21 | 0.089 |
| Previous sprain, n (%) | 121 (43.2) | 8 (28.6) | 0.195 |
| Baseline CAIT | 25.47 ± 3.81 | 25.04 ± 3.94 | 0.583 |
| Baseline CAIT ≤24, n (%) | 76 (27.1) | 7 (25.0) | 0.984 |
| YBT composite (%) | 91.53 ± 7.60 | 89.85 ± 9.16 | 0.357 |
| Eyes-closed COP velocity (mm/s) | 31.83 ± 8.90 | 32.76 ± 10.00 | 0.639 |
| Lateral forefoot pressure (kPa) | 276.99 ± 50.92 | 279.40 ± 51.84 | 0.815 |
| Medial-to-lateral ratio | 0.90 ± 0.16 | 0.89 ± 0.16 | 0.751 |
| Heel asymmetry (%) | 8.59 ± 4.66 | 9.45 ± 4.94 | 0.385 |

# Table S2. Outcome components, timing and side consistency

| **Item** | **Count / result** |
| --- | --- |
| Complete composite outcome | 107 |
| Complete outcome with new sprain during follow-up | 77 |
| Complete outcome without new sprain | 30 |
| Repeated CAIT component only | 14 |
| Giving-way/recurrent-sprain component only | 20 |
| CAIT plus event, symptoms ≤3 months | 5 |
| No component pattern | 162 |
| Baseline CAIT ≤24 among all participants | 83/308 (26.9%) |
| Baseline CAIT ≤24 among outcome-positive participants | 75/107 (70.1%) |
| Index assigned by unilateral previous sprain | 109 (development 101; holdout 8) |
| Index assigned by lower baseline CAIT | 198 (development 179; holdout 19) |
| Index assigned by non-dominant tie-break | 1 (development 0; holdout 1) |
| Bilateral previous sprain | 20 |
| Bilateral composite outcome | 8 |
| Outcome included index ankle | 107/107 |
| All primary measurements aligned to index ankle | 308/308 |
| Completed monthly assessments | 308/308 × 12 months |
| Censoring among analytic cohort | 0 |

# Table S3. Device and acquisition specifications

| **Domain** | **Equipment / acquisition** | **Trial structure** | **Processing** |
| --- | --- | --- | --- |
| Y-Balance Test | Standard YBT kit; index ankle; ASIS-to-medial-malleolus leg length | 3 practice; 3 formal trials/direction; 20-s rest | Maximum valid direction distance; standard composite formula |
| Static balance | AMTI OR6-7; 1000 Hz | 1 practice; 3 × 30 s eyes open and 3 × 30 s eyes closed; 60-s rest | 4th-order zero-phase Butterworth low-pass, 10 Hz; trial means |
| Plantar pressure | Novel emed-x; 100 Hz; 4 sensors/cm²; barefoot | 3 × 30-s quiet standing; 3 practice + 5 formal lunges; 60-s rest | Regional masks; five-trial lunge mean and three-trial standing mean |

# Table S4. Complete primary model specification

| **Term** | **Coding / standardisation** | **Coefficient** |
| --- | --- | --- |
| Intercept | — | −1.603628 |
| Previous lateral ankle sprain | 0 = no; 1 = yes | 1.230074 |
| YBT composite | Z = (value − 91.525714)/7.588235 | −0.775820 |
| Eyes-closed COP velocity | Z = (value − 31.831429)/8.885902 | 0.669609 |
| Lateral forefoot peak pressure | Z = (value − 276.985714)/50.826119 | 0.549979 |
| Medial-to-lateral load ratio | Z = (value − 0.904857)/0.163332 | −0.713223 |
| Heel contact-area asymmetry | Z = (value − 8.594286)/4.650215 | 0.477581 |

For an individual athlete, logit(p) = −1.603628 + 1.230074 × previous sprain − 0.775820 × ZYBT + 0.669609 × ZCOP + 0.549979 × Zlateral pressure − 0.713223 × Zmedial:lateral ratio + 0.477581 × Zheel asymmetry, and p = exp[logit(p)]/{1 + exp[logit(p)]}. This equation is supplied for reproducibility and is not recommended for unsupervised clinical deployment.

# Table S5. Domain, benchmark and sensitivity analyses

| **Analysis** | **Development AUC** | **Holdout AUC** | **Development Brier** | **Interpretation** |
| --- | --- | --- | --- | --- |
| History only | 0.678 | 0.639 | 0.199 | Lowest discrimination |
| Balance only | 0.881 | 0.834 | 0.113 | Objective balance domain |
| Plantar pressure only | 0.895 | 0.904 | 0.100 | AUC similar to combined |
| Six-predictor model | 0.892 | 0.850 | 0.080 | Primary model |
| Baseline CAIT alone | 0.951 | 0.941 | 0.085 | Best simple benchmark |
| CAIT plus previous sprain | 0.950 | 0.947 | 0.085 | LR vs CAIT P = 0.662 |
| CAIT plus six predictors | 0.952 | 0.963 | 0.086 | LR vs CAIT P = 0.940 |
| Baseline CAIT >24, refitted model | 0.680 | 0.603 | 0.099 | Dev CITL 0.000 (-0.426 to 0.426), slope 1.000 (0.545-1.455), O:E 1.00 (0.66-1.45); holdout CITL 0.249 (-0.943 to 1.442), slope 0.714 (-0.381 to 1.809), O:E 1.19 (0.32-3.04) |
| Unilateral-sprain index subgroup | 0.921 | 1.000 | 0.091 | Holdout n = 8; not interpretable |
| Six predictors plus training volume | 0.904 | 0.850 | 0.079 | LR vs primary P = 0.085 |
| Spline-enhanced model | 0.909 | 0.840 | 0.075 | Corrected AUC 0.894; no holdout gain |

# Table S6. Robustness, subgroup and missing-data analyses

| **Analysis** | **Result** |
| --- | --- |
| Leave-one-centre-out pooled performance | AUC 0.879; Brier 0.088; calibration intercept -0.069; slope 0.915 |
| Centre-specific AUC range | 0.807-0.979; evaluates predictive heterogeneity, not measurement equivalence |
| Centre fixed-effect likelihood-ratio test | P = 0.878 |
| Index-ankle assignment | Unilateral sprain 109; lower CAIT 198; non-dominant tie-break 1 |
| CAIT-independent index subgroup | n = 101; events = 54; five-predictor AUC 0.921 (95% CI 0.855-0.975); Brier 0.091 |
| Training-volume augmentation | OR per SD 1.43 (95% CI 0.95-2.15); P = 0.091; LR P = 0.085; development AUC 0.904; Brier 0.079 |
| Training-volume model: development calibration | CITL 0.000 (95% CI -0.392 to 0.392); slope 1.000 (0.778-1.222); O:E 1.00 (0.81-1.22) |
| Training-volume model: holdout calibration | CITL 0.096 (95% CI -1.195 to 1.387); slope 0.729 (0.235-1.223); O:E 1.02 (0.51-1.83); primary-model values 0.187, 0.789 and 1.04 |
| Previous-sprain coefficient after training adjustment | OR 3.42 to 3.53; relative change +3.3% |
| Lower-YBT coefficient after training adjustment | OR 2.18 to 2.14; relative change -1.7% |
| Higher-COP-velocity coefficient after training adjustment | OR 1.96 to 1.97; relative change +0.9% |
| Higher-lateral-pressure coefficient after training adjustment | OR 1.73 to 1.78; relative change +2.3% |
| Lower-medial: lateral-ratio coefficient after training adjustment | OR 2.04 to 2.07; relative change +1.2% |
| Higher-heel-asymmetry coefficient after training adjustment | OR 1.61 to 1.52; relative change -6.1% |
| Training-adjustment coefficient summary | Range of relative OR changes -6.1% to +3.3%; mean absolute change 2.6% |
| Body-mass augmentation | OR per SD 1.32 (95% CI 0.86-2.02); P = 0.201; LR P = 0.193 |
| BMI augmentation | OR per SD 1.43 (95% CI 0.95-2.15); P = 0.084; LR P = 0.081 |
| Nonlinearity tests | YBT P = 0.023; COP P = 0.261; lateral pressure P = 0.011; medial:lateral ratio P = 0.713; heel asymmetry P = 0.021 |
| Selected interactions | Previous sprain × YBT P = 0.505; × COP P = 0.805; × medial:lateral ratio P = 0.323 |
| Male subgroup | n = 168; events = 61; AUC 0.900; Brier 0.080 |
| Female subgroup | n = 140; events = 46; AUC 0.875; Brier 0.084 |
| Age <18 years | n = 96; events = 35; AUC 0.831; Brier 0.102 |
| Age ≥18 years | n = 212; events = 72; AUC 0.911; Brier 0.072 |
| Missing primary predictors | 0 for every primary predictor, baseline CAIT and training volume |
| Incomplete 12-month follow-up in analytic cohort | 0 |

# Table S7. Complete performance of the baseline CAIT benchmark

| **Cohort** | **AUC (95% CI)** | **Brier score (95% CI)** | **Calibration-in-the-large; slope; O:E** | **CAIT ≤24 sensitivity; specificity** |
| --- | --- | --- | --- | --- |
| Development (280/96) | 0.951 (0.926-0.971) | 0.085 (0.065-0.105) | 0.000 (-0.419 to 0.419); 1.000 (0.729-1.271); 1.00 (0.81-1.22) | 0.708 (0.611-0.790); 0.957 (0.917-0.978) |
| Geographic holdout (28/11) | 0.941 (0.845-1.000) | 0.092 (0.035-0.158) | 0.319 (-0.862 to 1.500); 1.280 (0.082-2.479); 1.08 (0.54-1.94) | 0.636 (0.354-0.848); 1.000 (0.816-1.000) |

# Table S8. Within-session repeatability of primary measurement variables

| **Primary measurement** | **Participants / trials** | **ICC(A,1)** | **Within-subject CV** |
| --- | --- | --- | --- |
| YBT trial-composite (%) | 308 / 3 | 0.994 | 0.7% |
| Eyes-closed COP velocity (mm/s) | 308 / 3 | 0.991 | 2.6% |
| Lateral forefoot peak pressure (kPa) | 308 / 5 | 0.964 | 3.5% |
| Medial-to-lateral load ratio | 308 / 5 | 0.982 | 2.5% |
| Heel contact-area asymmetry (%) | 308 / 3 | 0.957 | 11.4% |

# Table S9. Bootstrap coefficient stability across 2,000 complete refits

| **Predictor (adverse direction)** | **Bootstrap median OR** | **Bootstrap 95% interval** | **Adverse sign retained** |
| --- | --- | --- | --- |
| Previous sprain | 3.59 | 1.55-9.27 | 99.8% |
| Lower YBT composite | 2.24 | 1.25-4.49 | 99.7% |
| Higher eyes-closed COP velocity | 1.98 | 1.24-3.42 | 99.9% |
| Higher lateral forefoot pressure | 1.75 | 1.00-3.26 | 97.6% |
| Lower medial-to-lateral ratio | 2.11 | 1.44-3.38 | 100.0% |
| Higher heel asymmetry | 1.65 | 0.87-3.31 | 93.7% |

# Supplementary note on interpretation

The baseline-CAIT>24, index-selection, training-exposure, subgroup, nonlinear and interaction analyses were used to characterise limitations and robustness rather than to select the primary model. The geographic holdout and subgroup samples were too small for claims of equal or transportable performance. Within-session ICCs do not establish between-day, inter-rater or cross-centre measurement equivalence. Bootstrap coefficient stability describes the fitted prediction equation and does not establish causal or independently modifiable biomechanical mechanisms. The primary model remains a research model for the stated composite outcome and provides no demonstrated incremental clinical value beyond baseline CAIT.
