# Supplementary material for: Limited incremental value of balance and plantar-pressure measures beyond baseline CAIT for a 12-month composite ankle-instability outcome in competitive badminton players
Source: Front Physiol. 2026 Aug 28;17:1918241. doi: 10.3389/fphys.2026.1918241 (PMC13561929; doi:10.3389/fphys.2026.1918241)
Supplement: Supplementary file 2 [file Table1.docx]

Completed TRIPOD Checklist

Study: Development and Preliminary Geographic Evaluation of a Balance- and Plantar-Pressure Model for a 12-Month Composite Ankle-Instability Outcome in Competitive Badminton Players

This checklist maps the 27 main TRIPOD items to the revised manuscript. Detailed sub-item reporting is provided in the manuscript and supplementary material.

| **Item** | **Topic** | **Reporting content** | **Location** |
| --- | --- | --- | --- |
| 1 | Title | Identifies model development/evaluation, target population and outcome | Title |
| 2 | Abstract | Structured summary of objectives, data, participants, predictors, modelling, performance and limitations | Abstract |
| 3 | Background | Clinical context and rationale for the model | Introduction, paragraphs 1–4 |
| 4 | Objectives | Study objectives, target population, outcome and comparisons | Introduction, final paragraph |
| 5 | Source of data | Study design, dates, centres and cohort allocation | Methods 2.1 |
| 6 | Setting | Training-centre setting, eligibility and recruitment | Methods 2.1; Figure 1 |
| 7 | Participants | Eligibility, exclusions, consent and follow-up | Methods 2.1–2.2 |
| 8 | Outcome | Definition, timing, side, components and adjudication | Methods 2.2; Supplementary Table S2 |
| 9 | Predictors | Definitions, measurement timing, side and blinding information | Methods 2.3–2.5; Supplementary Table S3 |
| 10 | Sample size | Contemporary sample-size adequacy criteria and limitations | Methods 2.6 |
| 11 | Missing data | Amount of missingness and analysis approach | Methods 2.7; Supplementary Table S6 |
| 12 | Data preparation | Index ankle, trial aggregation, standardisation and coding | Methods 2.2–2.7; Supplementary Table S4 |
| 13 | Model development | Fixed six-predictor logistic model and complete fitting procedure | Methods 2.7 |
| 14 | Model specification | Intercept, coefficients, coding and standardisation constants | Results 3.3; Supplementary Table S4 |
| 15 | Model performance | AUC, Brier score, calibration and decision curves | Methods 2.7; Results 3.5; Figures 3–4 |
| 16 | Model evaluation | Geographic holdout and leave-one-centre-out evaluation | Methods 2.7; Results 3.5 |
| 17 | Heterogeneity/fairness | Centre, sex and age exploratory analyses | Results 3.5; Supplementary Table S6 |
| 18 | Open science | Data access, code, dictionary and model coefficients | Data availability statement; submission files |
| 19 | Patient/public involvement | No patient or public involvement in study design or reporting | Supplementary note / not applicable |
| 20 | Participant flow | Screening, exclusions, losses, cohort split and outcomes | Figure 1; Results 3.1 |
| 21 | Participant characteristics | Development characteristics and holdout comparison | Table 1; Supplementary Table S1 |
| 22 | Model development results | Full coefficient estimates and uncertainty | Table 3; Supplementary Table S4 |
| 23 | Model performance results | Apparent, corrected, holdout and centre-based performance | Table 4; Figure 4 |
| 24 | Additional analyses | Domain models, CAIT benchmark, sensitivity, nonlinearity and interactions | Results 3.4–3.5; Supplementary Tables S5–S6 |
| 25 | Interpretation | Principal findings with cautious predictive interpretation | Discussion, paragraphs 1–6 |
| 26 | Limitations | Outcome ambiguity, baseline CAIT, sample size, reliability, equipment and generalisability | Discussion, paragraphs 6–8 |
| 27 | Implications | Research-only status and need for independent evaluation and impact studies | Discussion and Conclusion |
